# Integrating viral hepatitis management into the emergency department: A further step towards viral hepatitis elimination

**DOI:** 10.1016/j.jhepr.2023.100932

**Published:** 2023-10-12

**Authors:** Jordi Llaneras, Juan Carlos Ruiz-Cobo, Ariadna Rando-Segura, Ana Barreira-Díaz, Raquel Domínguez-Hernández, Francisco Rodríguez-Frías, Magda Campins, Joan Colom, Miguel Angel Casado, Albert Blanco-Grau, Juan Bañares, Arnau Monforte, Anna Falcó-Roget, Lourdes Ruíz, Beatriz Meza, Tomàs Pumarola, Mar Riveiro-Barciela, Rafael Esteban, María Buti

**Affiliations:** 1 Emergency Department, Hospital Universitari Vall d’Hebron, Barcelona, Spain; 2 Liver Unit, Hospital Universitari Vall d'Hebron, Barcelona, Spain; 3 Microbiology Department, Hospital Universitari Vall d'Hebron, Barcelona, Spain; 4 CIBEREHD, Instituto de Salud Carlos III, Madrid, Spain; 5 Pharmacoeconomics & Outcomes Research Iberia (PORIB), Madrid, Spain; 6 Biochemistry Department, Hospital Universitari Vall d’Hebron, Barcelona, Spain; 7 Epidemiology Service, Hospital Universitari Vall d’Hebron, Barcelona, Spain; 8 Public Health Agency of Catalonia, Health Department, General Subdirection on Prevention, Control and Care on Addictions, HIV, STI and Viral Hepatitis, Barcelona, Spain; 9 Infectious Diseases Department, Hospital Universitari Vall d’Hebron, Barcelona, Spain

**Keywords:** HBV, HCV, HDV, Mass screening, Emergency service, Cost-effectiveness analysis

## Abstract

**Background & Aims:**

Many people with HCV and HBV infection are unaware of their condition, particularly at-risk and vulnerable populations who face barriers for screening and linkage to care. Emergency departments are often their only point of contact with the health system.

**Methods:**

This is a prospective study investigating HBsAg and HCV antibody testing, with reflex testing for HDV antibodies and HCV RNA, in adults attending an emergency department and requiring a blood test. Positive cases were linked to care. A cost-effectiveness analysis was performed.

**Results:**

From February 2020 to February 2022, a total of 17,560 individuals were screened. HBsAg was detected in 91 (0.5%), HCV RNA in 128 (0.7%), and HDV antibodies in two (0.01%) individuals. Nearly 40% of positive cases were unaware of their condition. Linkage to care was achieved in 42 of 56 HBsAg-positive and 45 of 69 HCV RNA-positive participants who were candidates for referral. HCV and HBV screening *vs*. no screening yielded 1.06 and 0.42 additional quality-adjusted life-years, respectively, with incremental cost–utility ratios of €7,629 and -€147 per quality-adjusted life-year gained, respectively, and proved even more cost-effective in patients with hepatitis C aged 40–70 years.

**Conclusions:**

On emergency department screening for hepatitis B, C, and D in Barcelona, the prevalence of HBsAg was 0.5% and HCV RNA 0.7%, approximately threefold higher than that observed in the general population. This strategy diagnosed patients with active HCV infection and no risk factors, who would not have been screened according to the current recommendations. Screening and linkage to care of viral hepatitis is cost-effective in this setting.

**Impact and implications:**

We evaluated the performance and cost-effectiveness of a viral hepatitis screening programme implemented in an emergency department, which aimed to identify and link to care people living with hepatitis B and C. Our findings reveal a threefold higher prevalence of hepatitis B and C than in the general Spanish population, possibly attributable to the role of the emergency department as the main healthcare gateway for vulnerable populations, who have a higher prevalence of viral hepatitis. Risk factors for viral hepatitis could not be identified in most people living with hepatitis B and C attending the emergency department; hence, screening beyond risk factors should be considered in hepatitis detection strategies. Emergency department screening is cost-effective for hepatitis C and is a cost-saving strategy for hepatitis B in our setting. These data should inform future updates to clinical guidelines.

## Highlights

• HCV/HBV prevalence at ED is threefold higher than that in the general population.
• Forty percent of people living with HCV or HBV were unaware of the infection.
• Risk factors for viral hepatitis were not identified in 60% of those with HCV and 92% of those with HBV.
• Screening for HCV and HBV in the ED is cost-effective.

## Graphical abstract

**Figure:**
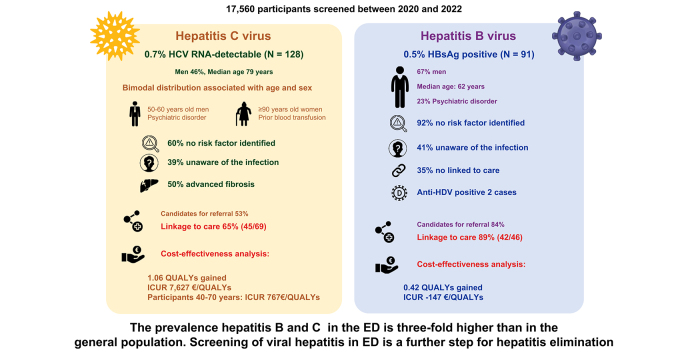

## Introduction

Hepatitis caused by chronic HCV and HBV infection is the leading cause of liver cancer worldwide.^1^^,^^2^ An estimated 1.1 million deaths in 2019 were related to complications of viral hepatitis.^3^

In 2015, the World Health Organization developed the Global Health Sector Strategy to eliminate viral hepatitis.^4^^,^^5^ The main goals of this initiative are a 90% reduction in the incidence of hepatitis B and C, and a 65% reduction in associated mortality by 2030. Mathematical models suggest that only 11 high-income countries (Australia, Canada, Denmark, Egypt, Finland, France, Georgia, Japan, Norway, Spain, and the UK) are on track to eliminate hepatitis C by 2030,^6^ whereas none of the participating countries are expected to eliminate hepatitis B by that time (Polaris Observatory, AASLD 2022).^7^ Better strategies to identify undiagnosed cases and ensure adequate follow-up are essential to achieve the World Health Organization goal. Micro-elimination approaches for at-risk vulnerable groups (*e.g.* people who inject drugs, immigrants, and prisoners) have been implemented in several countries as part of a comprehensive plan to reduce the number of people living with HCV and HBV infection and increase new diagnoses.^8^ Nonetheless, many of these groups have problems accessing healthcare by conventional circuits and are unaware that they have viral hepatitis.^9^ In addition, coinfection with HDV in HBV accelerates disease progression; therefore, HDV screening could be beneficial in all HBV-positive patients.^10^

Many countries have a viral hepatitis screening strategy based on risk factors,^11^ but interaction with healthcare centres is limited in vulnerable populations. Prisons, harm reduction centres, migrant shelters, and emergency departments (EDs) are often their only point of contact with the health system. In this line, the ED has been proposed as an effective setting for screening for blood-borne viruses and subsequent linkage to care.^12^^,^^13^ The aim of this study was to set up an ED screening and care linkage programme for viral hepatitis (B–D) and evaluate the cost-effectiveness of this initiative.

## Patients and methods

This is a prospective study conducted in the ED of Hospital Universitari Vall d’Hebron, a referral hospital serving the northern Barcelona health area with a catchment population of 450,000 inhabitants. The study was conducted from February 2020 to February 2022. Individuals aged ≥18 years attending the ED for a medical condition requiring a blood test were offered hepatitis B and C testing. HCV antibody (anti-HCV) and HBsAg were analysed in the same ED blood sample in all those who had not been tested for these markers in the previous 3 months. Reflex HCV RNA testing was automatically performed in anti-HCV-positive individuals, and reflex testing for HBV DNA and HDV antibodies (anti-HDV) in those testing HBsAg positive. Oral consent was obtained before testing.

The results obtained were encrypted and periodically sent to the study coordinator, in charge of review and validation. The coordinator, a hepatologist from the hospital Liver Unit, was responsible for referral to care. Medical records of participants with detectable HCV RNA or HBsAg were evaluated to determine their clinical, social, and functional status. After evaluation of each case, the study coordinator decided which patients were candidates for an outpatient consultation for evaluating HBV and HCV therapy and monitoring. On referral, liver fibrosis was evaluated by Fibrosis-4 (FIB-4), with advanced fibrosis defined by a FIB-4 score >3.25. Patients with treatment criteria were started on therapy at the first specialist visit to minimise the possibility of loss to follow-up.

### Study variables

Baseline demographics, epidemiologic variables, and relevant medical history (*e.g.* cardiovascular risk factors, previous liver disease, and psychiatric conditions) were obtained in all anti-HCV- or HBsAg-positive individuals. Medical histories were reviewed to collect previous data on their HCV/HBV/HDV serostatus.

Anti-HCV, HBsAg, and anti-HDV were determined using commercial tests (respectively, Elecsys anti-HCV II assay, Roche Diagnostics; Elecsys HBsAg II assay, Roche Diagnostics; and LIASON XL anti-HDV, Diasorin). HCV RNA was determined using the Cobas HCV test on a Cobas 6800 system (Roche Diagnostics), with a lower limit of detection of 10 IU/ml. HBsAg-positive patients were tested for HBeAg (Elecsys HBeAg, Roche Diagnostics) and HBV DNA (Cobas HBV test on a Cobas 6800 system, Roche Diagnostics). Information was collected on serious social problems and low life expectancy (<6 months) attributable to advanced age or a severe clinical condition, which would preclude referral of anti-HCV- or HBsAg-positive participants to the hepatology service. Cure of HCV infection was defined based on a sustained virological response (SVR) at week 12 after treatment completion.

### Cost-effectiveness analysis

Two lifetime Markov models (one each for hepatitis B and C) were used for the cost-effectiveness analysis, which compared viral hepatitis screening with no screening (Fig. S1). The target populations were HCV RNA-positive or HBsAg-positive participants considered for linkage to care. A previously described Markov model was adapted to simulate the clinical course of hepatitis C.^14^^,^^15^ A *de novo* model including parameters obtained from the literature^16^ was developed to project the course of hepatitis B. In both models, untreated patients progress according to the natural history of the disease. The perspective adopted in the analysis was the Spanish National Health Service, and only direct healthcare costs (screening, diagnosis, treatment, and disease management) were analysed. The screening programme was considered efficient if the incremental cost–utility ratio (ICUR) was below the willingness-to-pay threshold accepted in Spain: €25,000 per quality-adjusted life-year (QALY) gained.^17^ A discount rate of 3% was applied to health costs and outcomes.^18^

Sensitivity analyses were carried out based on the age groups with the highest prevalence of HCV (age 40–70 years) and HBV (age 50–80 years). A sensitivity analysis was performed including HCV treatment cost, with variations ranging from 30% to 60%.

### Statistical analysis

Variables with a normal distribution were compared using the Student *t* test and expressed as mean ± SD. Variables with a non-normal distribution were analysed using the Mann–Whitney *U* test and expressed as median and IQR. Categorical variables were compared using the Chi-square test or Fisher exact test when frequencies were <5% and expressed as frequency and percentage. Univariate logistic regression was performed to study associations with having detectable or undetectable HCV viraemia. A multivariate logistic regression model was developed including clinical and sociodemographic variables of interest to assess HCV and HBV infection, regardless of their significance in the univariate study. The most complete and parsimonious model was chosen. Odds ratios (ORs) and 95% CIs were calculated for independent predictors of having HBsAg or detectable HCV viral load. Data were analysed using StataCorp, 2015 (Stata Statistical Software, Release 14, StataCorp LP, College Station, TX, USA).

### Informed consent and institutional approval

The study was conducted in accordance with good clinical practice guidelines, was approved by the ethics committee of the referral hospital (PR[AG]86/2020), and had the support of the Subdirectorate General for Drug Dependency of the Public Health Agency of Catalonia. Patient consent for hepatitis screening was encouraged by iconographic resources in the form of posters and educational brochures displayed in patient-accessible areas of the ED. All data were anonymised and encrypted. All participants received oral, written, or iconographic information about the study before accepting participation.

## Results

### Prevalence of HBV, HCV, and HDV in the ED

Overall, 17,560 ED attendees requiring a blood test were screened between February 2020 and February 2022. Anti-HCV was detected in 684 (3.8%) participants, HCV RNA in 128 (0.7%), and HBsAg in 91 (0.5%). Anti-HDV were found in two (0.01%) individuals.

### Characteristics of participants with HCV

**Fig. 1:**
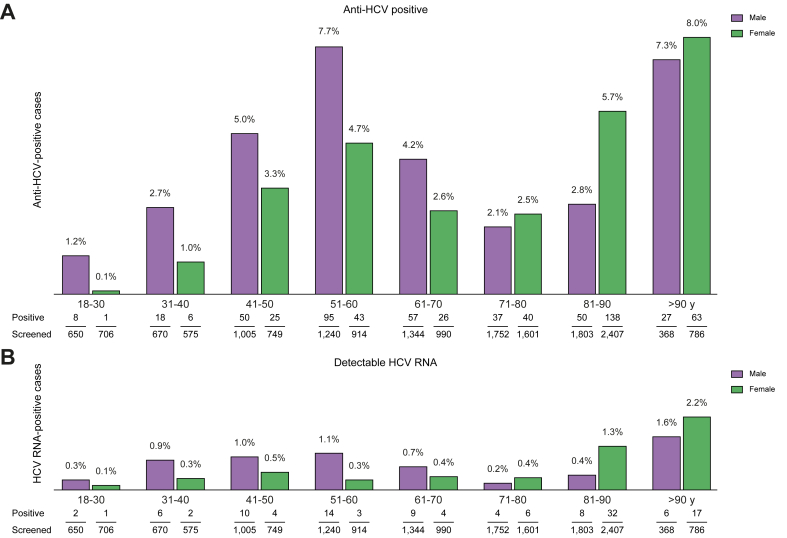
Prevalence of anti-HCV-positive and HCV RNA-positive cases according to sex and age of patients screened. (A) Anti-HCV-positive and (B) HCV RNA-positive cases.

The prevalence of anti-HCV and HCV RNA by participant age and sex is summarised in Fig. 1A and B. Both parameters showed a bimodal distribution associated with age. One peak was seen in men aged 50–60 years, mainly those with a history of injecting drug use (IDU) and psychiatric disorders. Another peak occurred in elderly participants (>90 years of age), with women predominating and a previous blood transfusion reported in 96%.

The main characteristics of participants with detectable anti-HCV and HCV RNA are shown in Table 1. Among 128 testing HCV RNA-positive, 46% were men, median age was 79 years, 94% were White, 26% reported current or former IDU, 4% had HIV coinfection, and 48 (38%) had a psychiatric condition. On univariate analysis, individuals with detectable HCV RNA had higher ALT values (17 *vs*. 29 IU/L) and FIB-4 score (2.23 *vs*. 3.3) than those without (*p* <0.05). Advanced fibrosis was documented in half of those (51%) with detectable and only 25% of those with undetectable HCV RNA (*p* <0.05). On multivariate analysis, IDU (OR 4.9) and transfusion history (OR 2.4) were independent risk factors for detectable HCV RNA (Table 1).

**Table 1:** Characteristics of patients with detectable anti-HCV on ED screening, and univariate and multivariate analysis of risk factors in relation to the HCV RNA detection.

	Anti-HCV positive (N = 684)	Undetectable HCV RNA (n = 556)	Detectable HCV RNA (n = 128)	Univariate analysis *p* value∗	Multivariate analysis	
					OR (95% CI)	*p* value
Men, n (%)	342 (50)	283 (51)	59 (46)	0.3		
Age (year), median (IQR)	72 (55–86)	71 (55–86)	79 (53–88)	0.3		
White, n (%)	630 (93)	510 (92)	120 (94)	0.4		
IDU (current or history), n (%)	125 (18)	92 (17)	33 (26)	**0.02**	**4.9 (2.3–10.5)**	**<0.001**
Alcohol consumption, n (%)	156 (22)	124 (22)	32 (25)	0.5		
Prior blood product transfusion, n (%)	45 (7)	30 (5)	15 (12)	**0.01**	**2.4 (1.2–4.8)**	**0.02**
Psychiatric disorder, n (%)	257 (38)	209 (38)	48 (38)	0.9		
HIV coinfection, n (%)	74 (11)	58 (10)	6 (4)	0.5		
Unaware of infected status, n (%)	270 (39)	226 (41)	44 (34)	0.1		
HBsAg, n (%)	5 (0.7)	5 (0.9)	0 (0)	0.4		
ALT (IU/L), median (IQR)	18 (12–30)	17 (12–27)	29 (17–56)	**<0.001**		
FIB-4, median (IQR)	2.31 (1.46–3.69)	2.23 (1.4–3.12)	3.3 (1.78–6)	**<0.001**		
Advanced fibrosis (FIB-4 >3.25), n (%)	206 (30)	141 (25)	65 (51)	**<0.001**		
Hepatocellular carcinoma, n (%)	26 (4)	16 (3)	10 (8)	**<0.001**		

Quantitative variables were expressed as medians (IQR) because of the non-normality of the distributions previously tested with a normality test (Shapiro–Wilks). To compare qualitative variables in univariate analysis, a Chi-square test was used, and Fisher's exact test was used in those expected frequencies <5. In the univariate comparison of quantitative variables, owing to non-normality, the Wilcoxon–Mann–Whitney *U* test was used. For the multivariate study, logistic regression was used, the independent variable being HCV RNA detectability or not. For all statistical studies, statistical significance was considered at *p* <0.05.

anti-HCV, HCV antibody; ED, emergency department; FIB-4, Fibrosis-4; IDU, injecting drug use.

∗ Comparison between patients with detectable and undetectable HCV RNA. P-values and ORs with statistical significance for a p-value of less than 0.05 are marked in bold.

HCV risk factors could not be identified in 77 (60%) of 128 individuals with detectable HCV RNA. The presence of at least one risk factor was more common in younger participants than in those older than 70 years; only 29% of individuals younger than 70 had no risk factors. Among 684 anti-HCV-positive cases, 220 individuals had been previously treated, and all except 11 achieved resolution of the infection. Of the 464 individuals who had apparently not been treated, 347 did not present viraemia, and spontaneous resolution of the infection was assumed.

Of the 128 HCV RNA-positive patients, 72 had been previously diagnosed with HCV (median time since diagnosis 2.63 years, IQR 0.97–5.18 years) although not all were aware of their HCV status. Only two of the 72 were correctly linked to care before their ED visit and could have received HCV treatment regardless of screening, 61 had no or very irregular follow-up, and nine had a very low life expectancy. In addition, 57 (44%) individuals with active infection were unaware of their condition. HCV infection was documented in the clinical records of 13 of these participants, but they had not been referred to a specialist.

### Characteristics of HBsAg-positive participants

Among the 91 HBsAg-positive individuals, most were men (67%), middle aged (62 years, IQR 49–76 years), and White (76%) (Table 2). Thirty-seven (41%) were unaware of their HBV infection, and 19 (35%) of the 54 who knew about the infection had not been linked to care. In 92% of HBsAg-positive individuals, no risk factor associated with the infection could be identified. Five HBsAg-positive participants had anti-HCV with undetectable HCV RNA.

**Table 2:** Characteristics of HBsAg-positive patients identified on ED screening.

	HBsAg positive (n = 91)
Male, n (%)	61 (67)
Age (years), median (IQR)	62 (49–76)
White, n (%)	69 (76)
IDU (current or history), n (%)	2 (2)
Alcohol consumption, n (%)	13 (14)
Prior transfusion of blood products, n (%)	4 (4)
Psychiatric disorder, n (%)	21 (23)
HIV coinfection, n (%)	1 (1)
Patients’ infection unawareness, n (%)	37 (41)
Anti-HCV, n (%)	5 (5)
ALT (IU/L), median (IQR)	22 (14–36)
FIB-4, median (IQR)	1.79 (1.04–2.9)
Late diagnosis (FIB-4 >3.25), n (%)	18 (20)
Hepatocellular carcinoma, n (%)	5 (6)
HBeAg, n (%)	4 (4)
Anti-HDV, n (%)	2 (2)

Quantitative variables were expressed as medians (IQR) because of the non-normality of the distributions previously tested with a normality test (Shapiro–Wilks).

anti-HCV, HCV antibody; anti-HDV, HDV antibody; ED, emergency department; FIB-4, Fibrosis-4; IDU, injecting drug use.

At the time of screening, 18 (20%) had advanced fibrosis and four (4%) had a diagnosis of hepatocellular carcinoma (HCC). Based on EASL criteria,^19^ 77% were classified as having HBeAg-negative chronic infection, 19% HBeAg-negative chronic hepatitis, and 4% HBeAg-positive chronic hepatitis. All 12 HBsAg-positive participants younger than 40 years were from countries without established HBV vaccination programmes (sub-Saharan Africa and Eastern Europe).

Two participants had HDV coinfection, one a native of Spain and the other from Eastern Europe. HDV RNA was undetectable in both cases, and neither had related risk factors.

### Linkage to care of participants with viral hepatitis

**Fig. 2:**
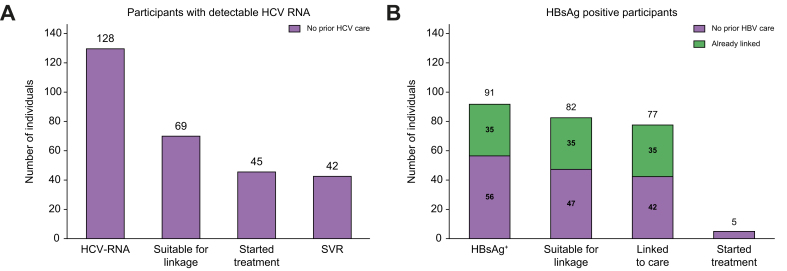
Linkage to care of participants testing HCV RNA-positive and HBsAg-positive on ED screening. (A) HCV RNA-positive and (B) HBsAg-positive on ED screening. ED, emergency department; SVR, sustained virological response.

Sixty-nine (54%) of 128 participants with detectable HCV RNA were considered eligible for linkage to a hepatologist (Fig. 2). The remaining 59 were excluded to linkage for the following reasons: life expectancy of <3 months in 40 (68%), serious social problem in 10 (17%), and death caused by the condition prompting their ED visit in nine (15%). Among the 69 referred, 45 (65%) started treatment, and all those who attended the 12-month follow-up visit (42 patients, 93%) achieved SVR. Twenty-four (35%) participants did not initiate treatment: 11 had social problems, seven serious comorbidities, two died owing to COVID-19 infection, and four did not attend the first visit.

Thirty-five (38%) of the 91 HBsAg-positive participants had been linked to care previously (Fig. 2). Forty-seven of the remaining 56 (84%) were considered eligible for linkage, and 42 (89%) were eventually linked; five individuals declined specialist referral. The reason for non-referral of nine cases was short life expectancy or severe social problems that prevented contact and referral. A flow chart depicting the screening and linkage results is shown in Fig. S2.

### Cost-effectiveness of ED screening for HCV and HBV

Implementation of an HCV screening programme *vs*. no strategy achieved an additional 1.06 QALY with an incremental cost of €8,110 per participant, yielding an ICUR of €7,629 per QALY gained (Table 3). In addition, compared with no screening, the HCV screening programme reduced the risk of developing decompensated cirrhosis by 67%, HCC by 60%, liver-related mortality by 62%, and liver transplant requirement by 63%, providing a €237,237 reduction in the associated cost of managing these complications.

**Table 3:** Results of HBV and HCV cost-effectiveness analysis per patient.

	ED screening programme	NO ED screening programme	Difference (screening *vs*. no screening)
**HCV screening in general population**			
LYG	11.43	10.85	0.58
QALYs	10.22	9.16	1.06
Total cost	€18,536	€10,426	€8,110
ICUR			**€7,629**
ICUR assuming 30% reduction in cost of treatment			**€4,187**
ICUR assuming 60% reduction in cost of treatment			**€679**

**HCV screening in population aged 40–70 years**			
LYG	18.47	16.81	1.66
QALYs	16.54	14.16	2.38
Total cost	€20,775	€18,953	€1,822
ICUR			**€767**
**HBV screening in general population**			
LYG	16.07	15.45	0.62
QALYs	1.73	0.71	1.02
Total cost	€3,274	€3,424	−€150
ICUR			**−€147**
**HBV screening in population aged 50–80 years**			
LYG	14.36	13.92	0.44
QALYs	0.99	0.57	0.42
Total cost	€2,599	€2,150	€449
ICUR			**€1,069**

The cost of diagnosing viral hepatitis included viral load testing; blood testing; tests to evaluate the degree of fibrosis or cirrhosis, if necessary; and outpatient specialist visits. Direct antiviral agents cost per patient with HCV was €17,126, and treatment cost per HBV patient per year has been reported.^20^ Healthcare costs associated with each health state were obtained from published studies^14^^,^^16^ and adjusted to 2021 prices using the Price Index of Consumption.

ED, emergency department; ICUR, incremental cost–utility ratio (additional cost of one QALY unit gained by one strategy compared with another); LYG, life-year gained; QALY, quality-adjusted life-year (a measure of health status that considers both the quantity and quality of life; one QALY is equivalent to 1 year in perfect health). The final ICUR result is marked in bold.

In comparison with no strategy, HBV screening led to a 0.42 QALY increase and a cost saving of €150 per participant, yielding an ICUR of −€147 per QALY gained and indicating a dominant strategy (Table 3). When compared with no screening, the screening programme averted a significant number of liver complications (decompensated cirrhosis decrease, 84%; HCC decrease, 78%) and reduced liver-related deaths by 82%, with an associated cost reduction of €220,385.

Sensitivity analyses showed that limiting HCV screening to the population aged 40 to 70 years decreased the ICUR to €791 per patient. In contrast, limiting HBV screening to those aged 50–80 years paradoxically increased the ICUR to €1,399. The screening age range did not have a significant impact on hepatic complications in either viral hepatitis, except for liver transplant in patients with HCV, where the percentage of averted procedures decreased from 63% in the overall population to 53% in the age-adjusted population.

## Discussion

This prospective study describes an integrated viral hepatitis screening and linkage-to-care programme for individuals attending a referral hospital ED and requiring a blood test. There was a threefold higher prevalence of hepatitis B and C in this setting than in the Spanish general population. In addition, 60% of those with hepatitis C and 92% with hepatitis B did not recognise having any risk factor for viral hepatitis, indicating that screening for this disease based on risk factors could miss a large percentage of people with these infections. Anti-HDV were detected in only two HBsAg-positive patients, resulting in an HDV prevalence of 2.2%, a lower value than has been previously reported^21^ but in line with more recent estimations.^22^

In-hospital screening allows retrieval of medical records to help determine whether patients should be referred to a specialist, and hospitals have the resources to contact and link patients to appropriate care. Among 69 HCV-positive candidates for referral, 45 (65%) were seen by a hepatologist and started treatment. All 45 participants completed treatment, and 42 (93%) are known to have achieved SVR. In view of the high efficacy of current HCV therapy, it is reasonable to assume that the three patients lost to follow-up also attained SVR. In addition to improving detection of chronic hepatitis, the cost-effectiveness analyses indicated that this ED screening approach could be an efficient strategy to incorporate into Spain’s national health system.

It is important to call to mind that 60% of HCV-positive participants in this study lacked associated risk factors. According to the Spanish Ministry of Health recommendations, these patients would not have been tested and diagnosed.^23^ The percentage was even higher in individuals with HBV infection: 92% lacked any known risk factors. This could be related to the stigma attached to risk factors and reluctance to report them, or to the lengthy interval between acquisition and diagnosis of the infection, which complicates their identification.^24^^,^^25^ These results suggest that HCV and HBV screening based on risk factors might hinder the diagnosis of these patients.

HCV infection had been recorded in the medical history of 23% (13/57) of participants with detectable HCV RNA, but they were unaware of their condition. Similar results were observed for HBV: 35% (19/54) had already been diagnosed before our screening and were lost to follow-up. Our data on lack of awareness and low linkage in hepatitis B infection concur with the findings from a previous ED screening study in Australia.^26^ Of particular note, half the individuals with active HCV infection and 18% of HBsAg-positive cases in our study had signs of advanced fibrosis at the diagnosis, highlighting the need for appropriate linkage to care.

Not all participants diagnosed in the ED were candidates for therapy. Only half of those with active HCV infection were referred to a specialist. This is partly because a large part were elderly patients with comorbidities and short life expectancy. Furthermore, 35% of those with active HCV and specialist referral did not attend follow-up. These were mainly people with significant social problems, thus supporting data on the difficulty of providing HCV treatment for certain populations.^27^ Forty-two of the 45 treated patients (93%) achieved SVR. In the HBsAg-positive group, 75% (42/56) of those lacking specialist follow-up were successfully linked to care. This aligns with the results of the study by Jacob *et al.*,^26^ in which more than 87% of patients were linked to care. We believe that the figure of a hepatologist acting as screening coordinator was a key factor facilitating fast and simple linkage to care, and reducing the time from diagnosis to treatment. This approach may be worth considering to increase the likelihood of specialist linkage from the ED.

The cost-effectiveness analysis showed that ED screening for HBV and HCV is efficient. As compared with no screening, the ICUR was below the willingness-to-pay threshold in Spain for HCV and was a dominant strategy for HBV. To our knowledge, this is the first data showing that ED screening for viral hepatitis in Spain is cost-effective and consistent with results from studies in the UK and France.^28^^,^^29^ The sensitivity analysis showed that HCV screening in patients aged 40–70 years (recommended by national scientific societies) was even more cost-effective, yielding a lower ICUR in the age cohort-adjusted scenario, as has been described in previous studies.^29^ In contrast, sensitivity analysis of the unvaccinated HBV population (age 50–80 years) showed a higher, but still very cost-effective, ICUR for ED screening.

ED testing for HCV and HBV is likely to be cost-effective in many other geographical areas depending on the prevalence of these infections. Further studies evaluating ED testing across different regions will be of help to inform testing guidelines.

### Limitations

The main limitation of this study relates to the viral hepatitis rates found in the ED-screened population compared with reported rates in Spain. Our cohort was drawn from all individuals attending the ED and requiring a blood test. However, elderly people with comorbidities consult most often in our hospital EDs, indicating selection bias. The onset of the SARS-CoV-2 pandemic during the study period may have magnified this situation, as a large number of elderly patients came to the ED at that time. The comparator data on HCV prevalence in the general Spanish population were taken from a study carried out in individuals younger than 80 years who volunteered for testing; the more vulnerable populations and elderly patients do not usually participate in this type of study. As to the HBV data, the prevalence study in Spain was based on an indirect registry of notifiable diseases, in which only acute hepatitis B cases were recorded. HBsAg reporting is not mandatory in asymptomatic patients; therefore, the data could underestimate the prevalence of HBsAg infection in the general population.

### Conclusions

Screening for viral hepatitis infection in ED attendees requiring a blood test showed a 0.7% prevalence of active HCV infection and an HBV prevalence almost three times higher than in the general population, despite an established vaccination programme. Forty percent of patients with HCV or HBV infection were unaware of their status, and a large percentage did not meet HCV/HBV screening criteria according to current recommendations. HCV/HBV screening in the ED and linkage to care is cost-effective.

## Financial support

We acknowledge funding from Gilead Sciences’ FOCUS programme to support screening and linkage to the first medical appointment after the diagnosis. The analysis was conducted without regard to specific products or treatments, if any. It is for the purpose of information alone and does not make or result in claims about any specific therapy. Gilead funding was used to finance the staff involved and the direct cost of laboratory testing, and to support the cost-effectiveness analysis of the screening strategy. The sponsor did not take part in the study design, data collection, interpretation of the data, writing of the report, or the decision to submit the paper for publication.

## Authors’ contributions

Planned and designed the study, and performed much of the pre-study research and conceptualisation: JL, JCR, AB, MB. Provided essential laboratory support and sample management: ARS, FRF, ABG, TP. Guided and supported the study from the public health perspective: MC, JC. Performed modelling of the cost-effectiveness analyses used to evaluate the study strategy: RDH, MAC. Wrote the first draft of the manuscript, except for the cost-effectiveness section: JL, JCR, MB. Wrote the cost-effectiveness section: RDH, MAB. Accessed and checked the data: JL, JCR, ARS. Contributed to the conduct of the study and refinement of the design as it progressed, and to revising, editing, and approving the final manuscript: all authors. Had full access to the study data and had the final responsibility of reviewing and accepting the manuscript for submission: all authors.

## Data availability statement

The data supporting the findings of this study are available upon reasonable request to the corresponding author.

## Conflicts of interest

JL has served as a speaker for Gilead and Pfizer. JCR has served as a speaker for and has received travel grants from Gilead. RDH is an employee of Pharmacoeconomics & Outcomes Research Iberia, a consultancy firm that has received unconditional funding from Gilead Sciences. FRF is a consultant, advisor, and speaker for Gilead. MAC is an employee of Pharmacoeconomics & Outcomes Research Iberia, a consultancy firm that has received unconditional funding from Gilead Sciences. MRB has received research educational and travel grants from Gilead and has served as a speaker for Gilead and GSK. RE has served as a speaker and advisor for Gilead and Abbvie. MB has served as a speaker and advisor for Gilead and Abbvie. All other authors declare no competing interests.

Please refer to the accompanying ICMJE disclosure forms for further details.
