# Supplementary material for: Integrating viral hepatitis management into the emergency department: A further step towards viral hepatitis elimination
Source: JHEP Rep. 2023 Oct 12;6(1):100932. doi: 10.1016/j.jhepr.2023.100932 (PMC10698271; doi:10.1016/j.jhepr.2023.100932)
Supplement: Multimedia component 1 [file mmc1.pdf]

Integrating viral hepatitis management into the emergency department: A further step towards viral hepatitis elimination

Jordi Llaneras, Juan Carlos Ruiz-Cobo, Ariadna Rando-Segura, Ana Barreira-Díaz, Raquel Domínguez-Hernández, Francisco Rodríguez-Frías, Magda Campins, Joan Colom, Miguel Angel Casado, Albert Blanco-Grau, Juan Bañares, Arnau Monforte, Anna Falcó, Lourdes Ruíz, Beatriz Meza, Tomàs Pumarola, Mar Riveiro-Barciela, Rafael Esteban, María Buti

Table of contents

Methods…………………………………………………………………………………2

Fig. S1……………………………………………………………………………………..3

Fig. S2……………………………………………………………………………………..5

Supplementary references………………………………………………………………6

Methods

A previously described Markov model was adapted to simulate the clinical course and costs of hepatitis C. [1, 2] A de novo model including parameters obtained from the literature [3] was developed to project the course and costs of hepatitis B. In both models, represented in Appendix Fig. S1, untreated patients progress according to the natural history of the disease. Patients enter the model based on median age (63 years for HBV infection and 73 years for HCV infection considering the patients who attend the hepatology outpatient clinic, and their fibrosis or cirrhosis status. The perspective adopted in the analysis was the Spanish National Health Service (NHS), and only direct health care costs (screening, diagnosis, treatment, and disease management) were analysed.

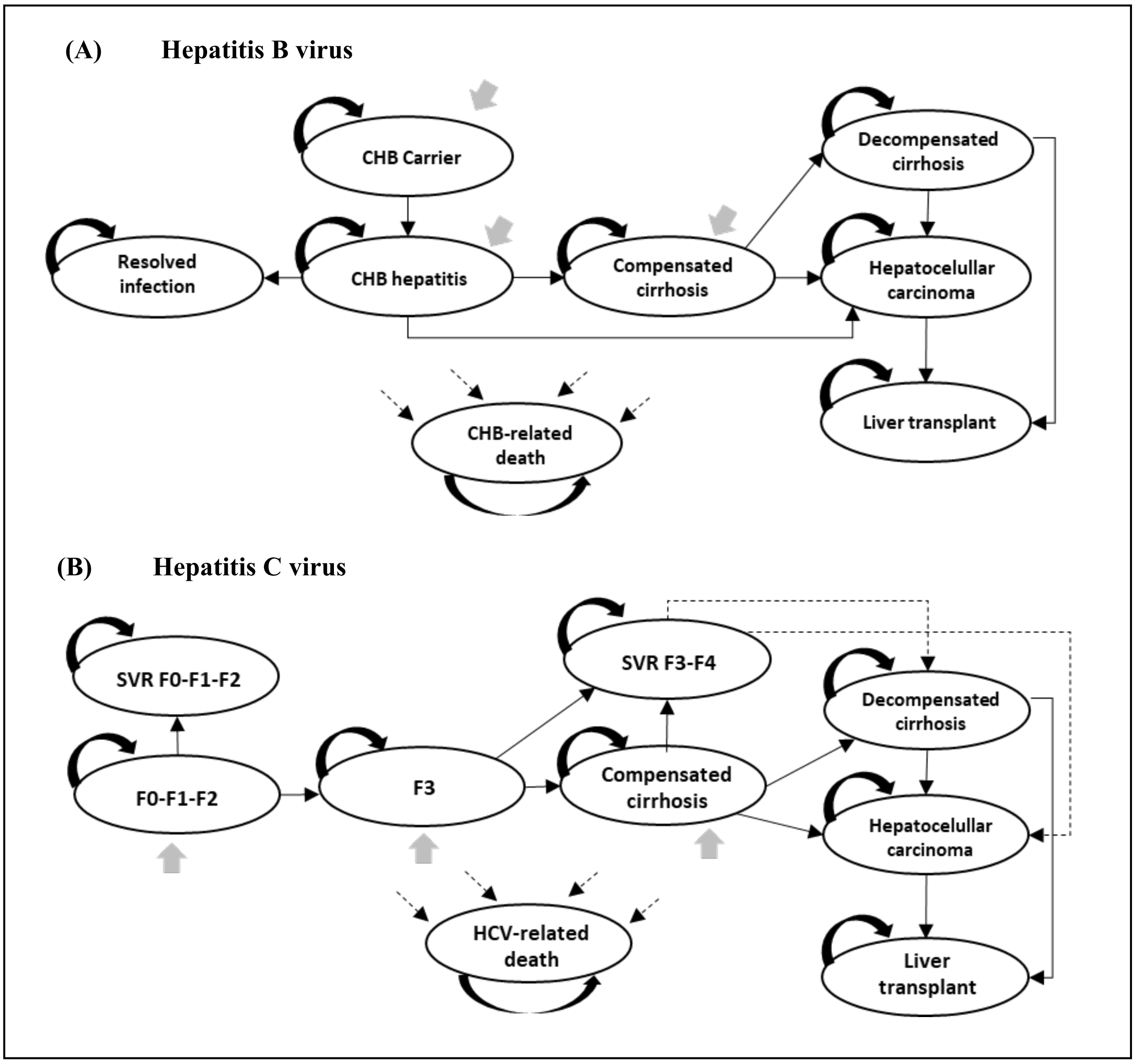

CHB, chronic hepatitis B; F0-F1-F2-F3, grades of liver fibrosis; HCV, hepatitis C virus; SVR, sustained virological response

Fig. S1. Markov model diagram for HBV (A) and HCV (B)

The models simulated the disease course through various health stages in the treated or untreated target population. The parameters required for each yearly cycle of the simulation (transition probabilities, utility values, and health status management costs) were those included in the previous model. Patients enter the model in the states where the arrows (⬆) are located for HBV (A) or HCV (B) and

move through the various mutually exclusive health states at the end of each annual cycle or remain in the same health state, with the exception of liver transplant (remains for only one cycle). (A) In HBV, chronic inactive carriers and those with resolved infection (1.5%) were also included. (B) HCV patients in SVR F0-F1-F2 are considered cured and remain in that state until their death, as patients with resolved infection.

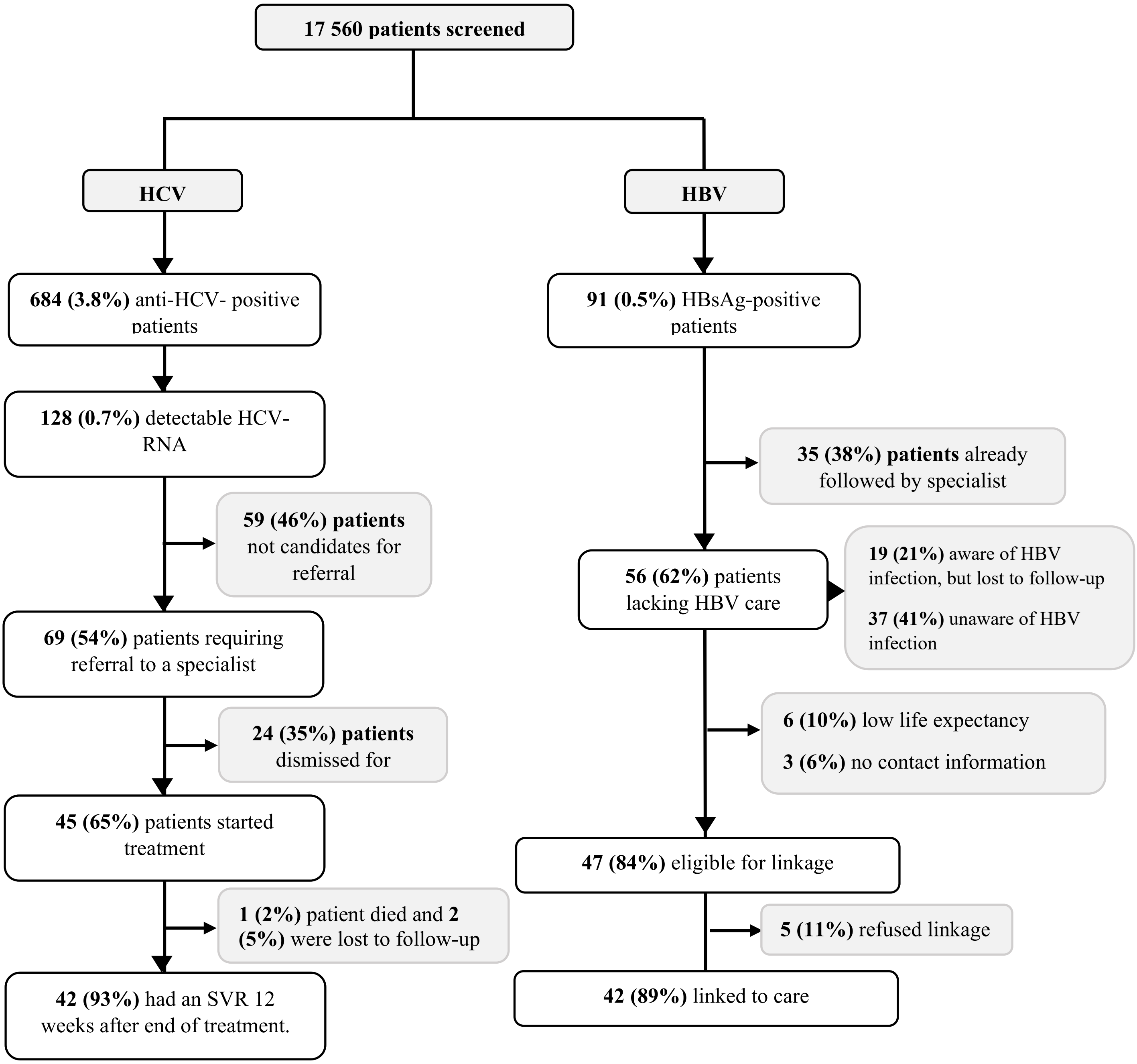

Fig. S2. Flow chart showing the results for ED screening and linkage to care of HBV- and HCV-positive individuals.

Supplementary references

1. Turnes J,. Domínguez-Hernández R, Casado MÁ. Value and innovation of direct-acting antivirals: long-term health outcomes of the strategic plan for the management of hepatitis C in Spain. Revista Española de Enfermedades Digestivas. 2017. DOI: 10.17235/reed.2017.5063/2017

2. Turnes J, Domínguez-Hernández R, Casado MÁ. Análisis coste-efectividad de dos estrategias de tratamiento para la hepatitis C crónica: antes y después del acceso a los agentes antivirales de acción directa en España. Gastroenterología y Hepatología. 2017;40(7):433–46. DOI: 10.1016/j.gastrohep.2017.05.004

3. Su S, Wong WC, Zou Z, Cheng DD, Ong JJ, Chan P, et al. Cost-effectiveness of universal screening for chronic hepatitis B virus infection in China: an economic evaluation. The Lancet Global Health. 2022;10(2):e278–87. DOI: 10.1016/S2214-109X(21)00517-9
